# Supplementary material for: Creation of acoustic vortex knots
Source: Nat Commun. 2020 Aug 7;11:3956. doi: 10.1038/s41467-020-17744-x (PMC7414909; doi:10.1038/s41467-020-17744-x)
Supplement: Supplementary file 1 — Supplementary Information [file 41467_2020_17744_MOESM1_ESM.pdf]

Supplementary Information for:
Creation of acoustic vortex knots

Hongkuan Zhang, Weixuan Zhang and Yunhong Liao et al.

Supplementary Note 1. The construction of torus knot functions based on the algebraic topology

The mathematical function with helical zero-value braids in the complex plane can be expressed as:

$$Q_{\mathrm{helix}}(u,v)=u^2-v^n \tag{1}$$

where u and v are complex variables. n is the number of repeats of the basic crossing sequence. The polynomial $Q_{\mathrm{helix}}(u,v)$ corresponds to the torus knots. Here, Hopf link (trefoil knot) belongs to polynomial $Q_{\mathrm{helix}}(u,v)$ with n=2 (n=3). To construct the knot function in 3D spaces, we should convert the 2D-complex polynomial $Q_{\mathrm{helix}}(u,v)$ into 3D-real space by making u and v be a function as r=(x, y, z). The relation between (u,v) and (x, y, z) can be expressed as:

$$u=[(r^2-1)+2\mathrm{i}z]/(r^2+1), \qquad v=2(x+\mathrm{i}y)/(r^2+1) \tag{2}$$

In this case, based on the coordinate transformation, Supplementary Equation (1) can be transformed into the (x, y, z) space as $C_{\mathrm{helix}}(x,y,z)$[1, 2]. Then, the field, which satisfies the paraxial equation and also concides with z=0 plane of $C_{\mathrm{helix}}(x,y,z)$ can be obtained as[1, 2]:

$$f_{\mathrm{Hopf}}(x,y,z)=[1-2R^2-4R^2e^{\mathrm{i}2\varphi}+R^4]-4iz(1-2R^2)-8z^2, \tag{3}$$

$$f_{\mathrm{trefoil}}(x,y,z)=[(R^4-1)(R^2-1)-8R^3e^{\mathrm{i}3\varphi}]+2i(9R^4-4R^2-1)z-8(9R^2-1)z^2-48iz^3 \tag{4}$$

where Supplementary Equation (3) corresponds to the case with n=2 and Supplementary Equation (4) corresponds to the case with n=3. Here, $R^2=x^2+y^2$ and $R\mathrm{e}^{\mathrm{i}\varphi}=x+\mathrm{i}y$ with x and y referring to the normalized coordinate values along the x- and y-axis. The nodal sets in Supplementary Equation (3) possesses the Hopf link topology and Supplementary Equation (4) possesses the trefoil knot nodal line.

Supplementary Note 2. Decoupled modulation of phase and amplitude for each pixel of initial wavefront to generate the acoustic vortex knots.

Different from the phase-only diffractive holographic scheme used in the optical system, here, each pixel of the initial wavefront (corresponds to a single unit of the metamaterials) should possess the ideal value of both phase and amplitude to generate acoustic vortex knot. In this condition, the knotted acoustic fields can be created in the transmission domain adjoined to the metamaterial. Here, we focus on the case of the Hopf link vortex line. The condition of the trefoil knot vortex line is identical. To generate the unbroken Hopf link vortex

loop, here, we should embed an algebraic knot function at $z=z_{Hopf}$ plane [$f_{Hopf}=(x, y, z=z_{Hopf})$] into the initial wavefront and then propagating the field. The suitable value of z_{Hopf} is limited by two factors: (1) The Hopf link nodal line of the knot function Supplementary Equation (3) should exist within the range from $-z_{Hopf}$ to z_{Hopf}. Only in this case, the unbroken Hopf vortex line can be realized. (2) The value of z_{Hopf} cannot be too large. This is to ensure that the acoustic vortex knots can be created with the propagation of the 2D initial wavefront. In our experiments, the value of z_{Hopf} is chosen as -0.8. Based on the same limitation, the value of $z_{trefoil}$ is chosen as -0.48.

Similarly, for the generation of the physically acoustic fields based on the knot function, the Gaussian profile $exp(-R^2/2W^2)$ should also be multiplied to the knot function. In this case, the explicit expressions of the acoustic field, where the vortex loops form Hopf link and trefoil knot, at the initial wavefront plane can be described by:

$$\psi_{Hopf}=f_{Hopf}(x,y,z=z_{Hopf})exp[-(x^2+y^2)/2W_{Hopf}^2] \quad (5)$$

$$\psi_{trefoil}=f_{trefoil}(x,y,z=z_{trefoil})exp[-(x^2+y^2)/2W_{trefoil}^2] \quad (6)$$

where the W_{Hopf} and $W_{trefoil}$ represent the normalized widths of the proposed Gaussian profile for the vortex structures of Hopf link and trefoil knot, respectively. Here, we select $W_{Hopf}=1.6$ and $W_{trefoil}=1.2$, respectively. The normalized values of x and y are both ranging from -5 to 5.

Supplementary Note 3. Phase-only diffractive holographic scheme for the generation of optical vortex knots

Both Hopf link and trefoil knot optical vortex lines can be generated by simply embedding an algebraic knot function at $z=0$ plane [$f_{Hopf}(x, y, z=0)$ and $f_{trefoil}(x, y, z=0)$] into the waist of a Gaussian beam and then propagating the field[1]. It is worthy to note that for the generation of the physical optical fields based on the knot function, the Gaussian profile $exp(-R^2/2W^2)$ should be multiplied to the knot function. In this case, the explicit expressions of the optical field, where the vortex loops are in the form of Hopf link and trefoil knot, at the waist plane can be described by:

$$\psi_{Hopf}=f_{Hopf}(x,y,z=0)exp[-(x^2+y^2)/2W_{Hopf}^2] \quad (7)$$

$$\psi_{trefoil}=f_{trefoil}(x,y,z=0)exp[-(x^2+y^2)/2W_{trefoil}^2] \quad (8)$$

The W_{Hopf} and W_{trefoil} represent the normalized widths of the proposed Gaussian profile for the vortex structures of Hopf link and trefoil knot, respectively. The transverse ranges along x- and y-directions are both ranging from -5 to 5. It is important to note that the values of W_{Hopf} and $W_{Trefoil}$ are crucial in the formation of the desired vortex topology[1].

To embed the Supplementary Equation (7) and Supplementary Equation (8) into the waist of a Gaussian beam, the diffractive holographic scheme based on the phase-only metasurface hologram can be used. The phase-only holograms can be modified to control not only the phase structure of the diffracted beams but their intensity[1-3]. Here, we focus on the case of the Hopf link vortex line. The condition of the trefoil knot vortex line is identical. Firstly, the phase distribution of the linked vortex field in the z=0 plane is calculated as $\phi_{\text{link}}(x,y) = \text{Arg}[\Psi_{\text{Hopf}}(x,y)]$. Then, the proposed phase distribution is added with a blazed diffraction grating $\phi_{\text{Blazed}}(\text{x},\text{y})$. In this condition, the first-order diffracted energy is angularly separated from the other orders. Finally, the desired intensity of the link beam in the z=0 plane $I_{\text{Hopf}}(\text{x},\text{y}) = \text{Abs}[\Psi_{\text{Hopf}}(x,y)]^2$ is also calculated and applied as a multiplicative mask to the phase distribution of the hologram, acting as a selective beam attenuator imposing the necessary intensity distribution on the first-order diffracted beam. Consequently, the phase distribution of the hologram to generate the Hopf link vortex line can be expressed as:

$$\phi_{\text{hologram}}(x,y)=\{[\phi_{\text{link}}(x,y) + \phi_{\text{Blazed}}(\text{x},\text{y})]_{\text{mod}2\pi} - \pi\}sinc[\pi - \pi I_{\text{Hopf}}(x,y)] + \pi \quad (9)$$

It is important to note that the designed phase-only hologram should possess a large number of sub-pixels (about 300×300) where the smooth phase modulation induced by the blazed grating can be realized. Mapping this scheme into the acoustic wave, the overall size of samples and generated vortex knots should both be extremely large. In this case, it is very difficult to observe the acoustic vortex fields experimentally.

Supplementary Note 4. Optimizing the number of pixels to decrease the transverse size of knot metamaterials

In principle, the larger the pixel number is, the better is the knotted vortex performance. Here, the optimization design is conducted to determine the minimal number of pixels (the lower limit) required for the metasurface-based hologram to generate the acoustic vortex knot, while keeping the unit cell size unchanged. To get the lower limit, we numerically calculated

the acoustic vortex knot generated by the aperture with different sets of pixel number for the creation of Hopf link and Trefoil knot vortex lines, as shown in Supplementary Figures 1 and 2, respectively. As the cell number decreases, the Hopf link and Trefoil knot vortex lines deform in size and shape, yet maintain topologically invariant. It is found that the Hopf link (Trefoil knot) vortex line disappears when the number of pixels for the metasurface-based hologram is reduced to less than 12×12 (16×16), which corresponds to the pixel limitation of metasurface to generate vortex knots. Below this limit, the topological phase transition of the vortex line (broken and reconnection) appears. In our study, a moderate pixel number 24×24 that lies above the lower limit is chosen, which is adequate to our needs for the vortex knot demonstration.

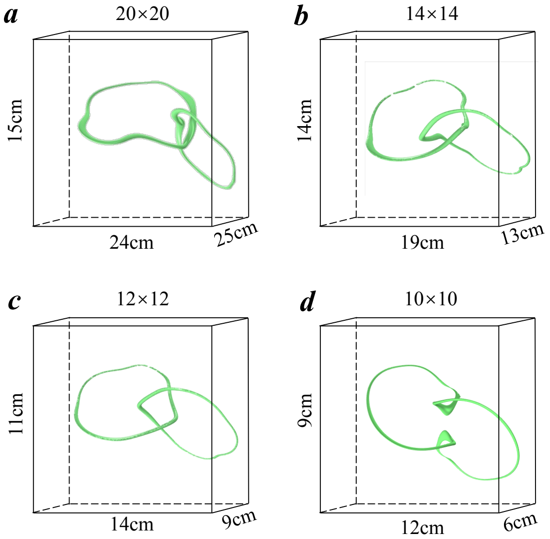

Supplementary Figure 1. (a)-(d) The numerical results of the vortex lines generated by the Hopf link related metasurface with 20×20, 14×14, 12×12, and 10×10 cells.

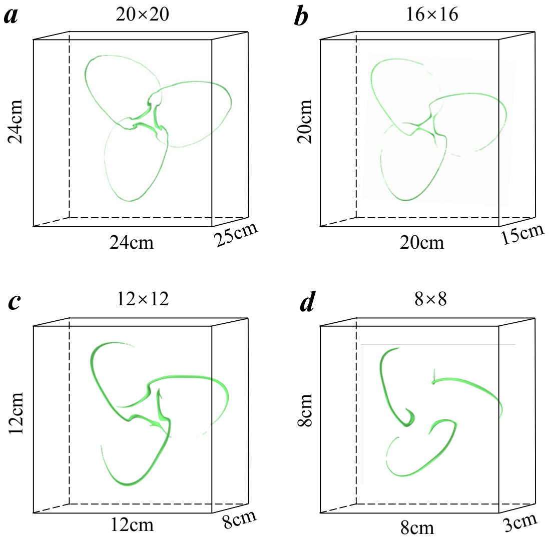

Supplementary Figure 2. (a)-(d) The numerical results of the vortex lines generated by the trefoil knot related metasurface with 20×20, 16×16, 12×12, and 8×8 cells.

Supplementary Figures 3 and 4 show the simulation result of the pressure amplitude and phase distribution at transverse-planes with different distances from the initial wavefront plane for the Hopf link and trefoil knot, respectively. It can be seen that the dark point with a local minimum pressure corresponds very well to the vortex point with phase singularities. By connecting the dark points on the different planes, an isolated acoustic Hopf link and trefoil knot vortex loop can be created, as shown in Supplementary Figures. 3c and 3f.

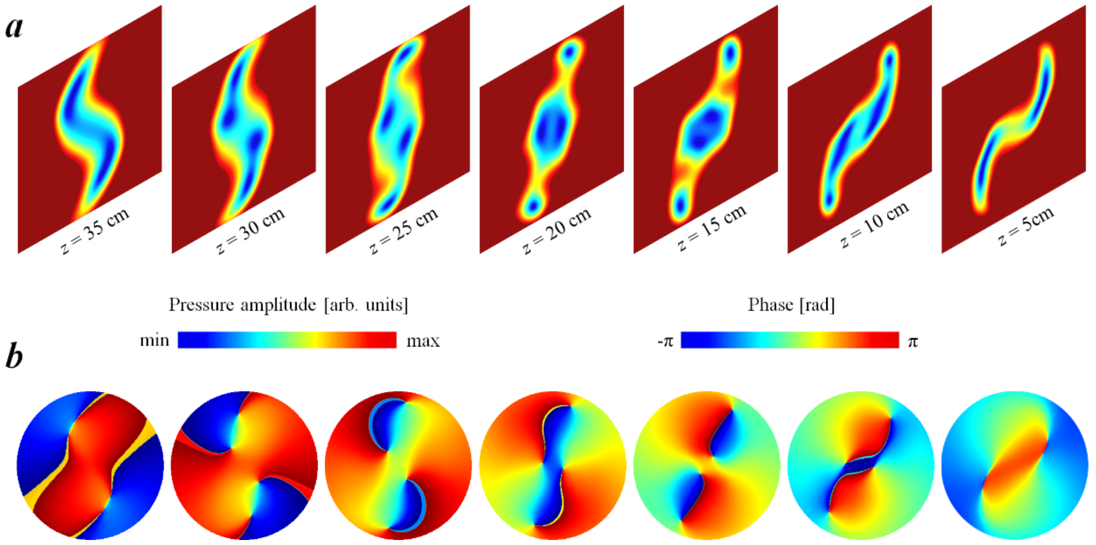

Supplementary Figure 3. Simulation results of (a) amplitude and (b) phase distributions of acoustic pressure at different *z*-plane for the Hopf link.

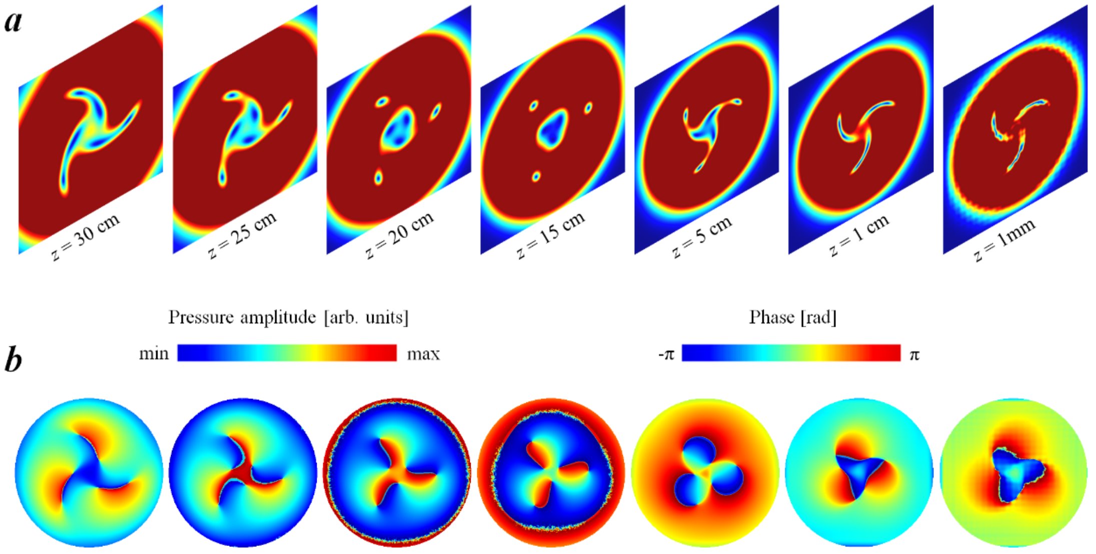

Supplementary Figure 4. Simulation results of (a) amplitude and (b) phase distributions of acoustic pressure at different *z*-plane for the trefoil knot.

Supplementary References

[1] M. R. Dennis, R. P. King, B. Jack, K. O'Holleran, M. J. Padgett, Nat. Phys. 2010, 6, 118–121.

[2] R. P. King, Knotting of optical vortices. University of Southampton, Applied Mathematics, Doctoral Thesis.

[3] J. Leach, M. R. Dennis, J. Courtial, M. J. Padgett, New J. Phys. 2005, 7, 55.
